# Multiresolution Aggregation Transformer UNet Based on Multiscale Input and Coordinate Attention for Medical Image Segmentation

**DOI:** 10.3390/s22103820

**Published:** 2022-05-18

**Authors:** Shaolong Chen, Changzhen Qiu, Weiping Yang, Zhiyong Zhang

**Affiliations:** School of Electronics and Communication Engineering, Sun Yat-sen University, Shenzhen 518000, China; chenshlong@mail2.sysu.edu.cn (S.C.); qiuchzh@mail.sysu.edu.cn (C.Q.); yangwp23@mail2.sysu.edu.cn (W.Y.)

**Keywords:** transformer, UNet, medical image segmentation, multiscale input, coordinate attention

## Abstract

The latest medical image segmentation methods uses UNet and transformer structures with great success. Multiscale feature fusion is one of the important factors affecting the accuracy of medical image segmentation. Existing transformer-based UNet methods do not comprehensively explore multiscale feature fusion, and there is still much room for improvement. In this paper, we propose a novel multiresolution aggregation transformer UNet (MRA-TUNet) based on multiscale input and coordinate attention for medical image segmentation. It realizes multiresolution aggregation from the following two aspects: (1) On the input side, a multiresolution aggregation module is used to fuse the input image information of different resolutions, which enhances the input features of the network. (2) On the output side, an output feature selection module is used to fuse the output information of different scales to better extract coarse-grained information and fine-grained information. We try to introduce a coordinate attention structure for the first time to further improve the segmentation performance. We compare with state-of-the-art medical image segmentation methods on the automated cardiac diagnosis challenge and the 2018 atrial segmentation challenge. Our method achieved average dice score of 0.911 for right ventricle (RV), 0.890 for myocardium (Myo), 0.961 for left ventricle (LV), and 0.923 for left atrium (LA). The experimental results on two datasets show that our method outperforms eight state-of-the-art medical image segmentation methods in dice score, precision, and recall.

## 1. Introduction

At present, more than 90% of medical data comes from medical images (magnetic resonance imaging (MRI), computed tomography (CT), etc.). The segmentation and subsequent quantitative evaluation of interested organs in medical images provide valuable information for pathological analysis. It is important for the planning of treatment strategy, the monitoring of disease progress, and the prediction of patient prognosis [1,2,3]. When doctors make a diagnosis, they usually first manually segment the organizations of interest in medical images and then perform quantitative and qualitative evaluations [4,5]. These tasks greatly increase the workload of doctors, cause doctors to be overloaded, and affect diagnostic effect. Therefore, it is urgent to study the automatic segmentation method of medical images to reduce the workload of doctors.

In the past decades, researchers have conducted a great amount of research on automatic segmentation of medical images, and many segmentation methods such as statistical shape models [6,7,8], anatomical atlases [9], and ray-casting [10] have been proposed. However, most of these traditional methods have problems such as complex design, poor versatility, and low segmentation accuracy. In recent years, deep learning has been widely used in medical image segmentation [11,12,13,14,15,16] and has achieved great success, especially the U-shaped and skip-connection based on convolution (UNet) [17], because it combines low-resolution information (providing the basis for object category recognition) and high-resolution information (providing the basis for precise segmentation and positioning), which is suitable for medical images segmentation. Then, researchers improved on the basis of UNet and proposed many better medical image segmentation methods [18,19,20,21,22,23] such as Att-UNet [18], Dense-UNet [19], R2U-Net [20], UNet++ [21], AG-Net [22], and UNet3+ [23]. However, due to the local characteristics of the convolution operation, the convolutional neural networks (CNN) can extract the detailed information of the image well, but there are limitations in extracting the global features. Therefore, it is difficult for the convolutional-based UNet to deal with long-range and global semantic information. In medical images, each organization is highly correlated, and the segmentation network needs to have strong global feature extraction capability.

In order to solve the problem of convolutional neural network in extracting global features, research scholars proposed transformer [24], which can extract the global characteristics of images well. Vision transformer (ViT) [25] is the first method to apply transformer to computer vision and has achieved superior performance. Subsequently, some researchers put forward many improved methods based on ViT, such as DeepViT [26], Cait [27], CrossViT [28], CvT [29]. Recently, some researchers have tried to combine transformer with UNet to improve the performance of UNet. Chen et al. proposed TransUNet [30]. This is the first time that transformer and UNet are combined, and good results have been achieved in the field of medical image segmentation. Subsequently, research scholars proposed more method combining transformer and UNet, such as, Swin-UNet [31], UNETR [32], UCTransNet [33], nnFormer [34]. However, existing transformer-based UNet methods do not comprehensively explore multiscale feature fusion, and there is still much room for improvement. Additionally, to the best of our knowledge, existing transformer-based UNet methods have not studied information aggregation of multiresolution input images.

In this paper, we propose a novel multiresolution aggregation transformer UNet (MRA-TUNet) based on multiscale input and coordinate attention for medical image segmentation. First, a multiresolution aggregation module (MRAM) is used to fuse the input image information of different resolutions, which enhances the input features of the network. Second, an output feature selection module (OFSM) is used to fuse the output information of different scales to better extract coarse-grained information and fine-grained information. We try to introduce a coordinate attention (CA) [35] structure for the first time to further improve the segmentation performance. We compare with state-of-the-art medical image segmentation methods on the automated cardiac diagnosis challenge (ACDC, https://acdc.creatis.insa-lyon.fr/ (accessed on 2 May 2022) [36]) and the 2018 atrial segmentation challenge (2018 ASC, http://atriaseg2018.cardiacatlas.org/ (accessed on 2 May 2022) [37]). Our method achieved average dice score of 0.911 for right ventricle (RV), 0.890 for myocardium (Myo), 0.961 for left ventricle (LV), and 0.923 for left atrium (LA). The experimental results on two datasets show that our method outperforms eight state-of-the-art medical image segmentation methods in dice score, precision, and recall.

Contributions:

1. A novel multiresolution aggregation transformer UNet (MRA-TUNet) based on multiscale input and coordinate attention for medical image segmentation is proposed. To the best of our knowledge, MRA-TUNet is the first transformer-based UNet method to study information aggregation of multiresolution input images.
2. MRA-TUNet is the first method to introduce coordinate attention structure in medical image segmentation.
3. MRA-TUNet outperforms the existing eight excellent medical image segmentation methods in dice score, precision, and recall, on the ACDC and the 2018 ASC.

## 2. Approach

**Figure 1:**
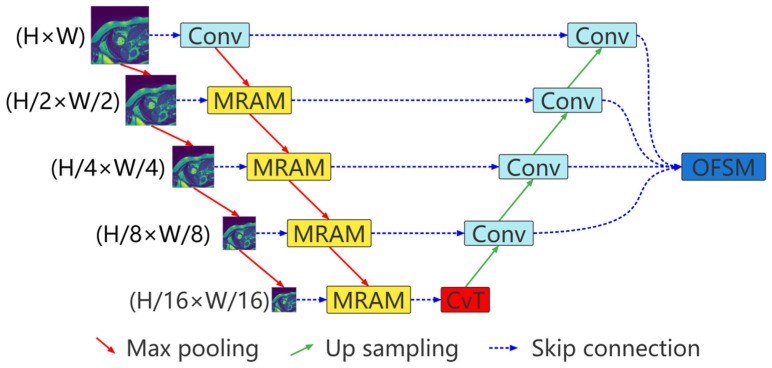
Multiresolution aggregation transformer UNet (MRA-TUNet). Conv: convolution block. MRAM: multiresolution aggregation module. CvT: convolution to vision transformer. OFSM: output feature selection module. H: image height. W: image width.

The proposed multiresolution aggregation transformer UNet (MRA-TUNet) is shown in Figure 1. It is mainly composed of multiresolution aggregation module (MRAM), convolution to vision transformer (CvT), and output feature selection module (OFSM). In Section 2.1, we introduce the proposed multiresolution aggregation module (MRAM). We introduce how to encode images with CvT in Section 2.2. In Section 2.3, we introduce the proposed output feature selection module (OFSM).

### 2.1. Multiresolution Aggregation Module

**Figure 2:**
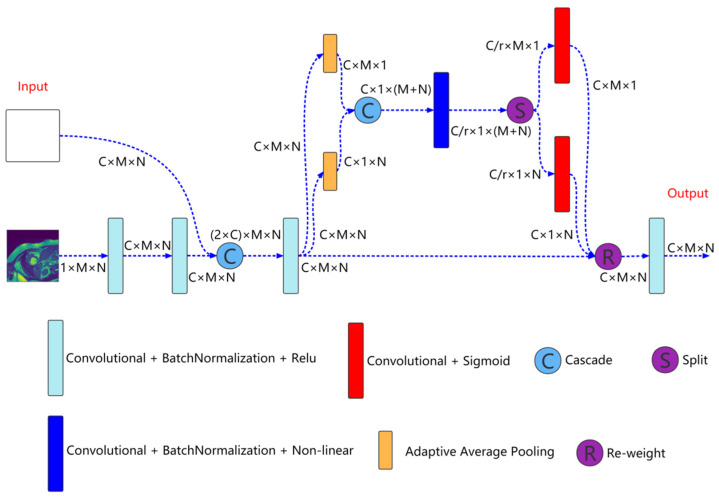
Multiresolution aggregation module structure. $M=H/2^{n}$, $N=W/2^{n}$, *C*, and *r* represent the number of channels and reduction rate, respectively.

Multiresolution aggregation module is shown in Figure 2, which is mainly used to fuse input image information of different resolutions to enhance the input characteristics of the network. As shown in the Figure 2, the inputs to the module are the current resolution image and the features come from the previous convolution unit. First, the feature of the current resolution image is extracted through two concatenated convolution units and cascade this feature with the feature come from the previous convolution unit. The expression is as follows:

(1) $$x_{n}^{c}=Cascade\left(F_{n-1},\;f(I_{n}\right))$$

Here, $x_{n}^{c}$ is the feature after cascade of the *n*th layer (*n* = 1, 2, 3, 4). Cascade() is the cascade operation. $F_{n-1}$ is the feature come from the previous convolution unit. $I_{n}$ represents the current resolution image. f() represents two concatenated convolution blocks.

Then, the cascaded feature $x_{n}^{c}$ is input to the coordinate attention for aggregation,

(2) $$x_{n}^{CA}=CA\left(x_{n}^{c}\right)$$

Here, $x_{n}^{CA}$ is the aggregated feature. CA() is the coordinate attention.

Finally, $x_{n}^{CA}$ is input to a convolution unit for feature extraction to obtain enhanced input feature,

(3) $$x_{n}^{EI}=Convolution\left(x_{n}^{CA}\right)$$

Here, $x_{n}^{EI}$ is the enhanced input feature. Convolution() is the convolution operation.

### 2.2. CvT as Encoder

**Figure 3:**
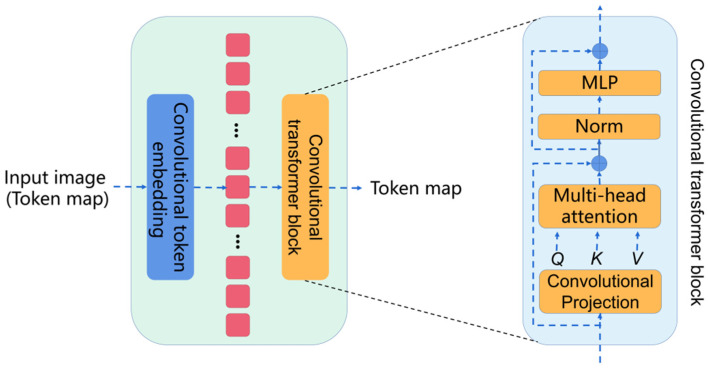
The basic module of the CvT.

Convolutional vision transformer (CvT) introduces convolutions into the vision transformer. The basic module of the CvT is shown in Figure 3, which is mainly composed of two parts:

Convolutional token embedding layer. The convolutional token embedding layer encodes and reconstructs the input image (2D reshaped token maps) as the input of the convolutional transformer block.

Convolutional transformer block. The convolutional transformer block uses depth-wise separable convolution operation for query, key, and value embedding, instead of the standard positionwise linear projection in ViT.

### 2.3. Output Feature Selection Module

**Figure 4:**
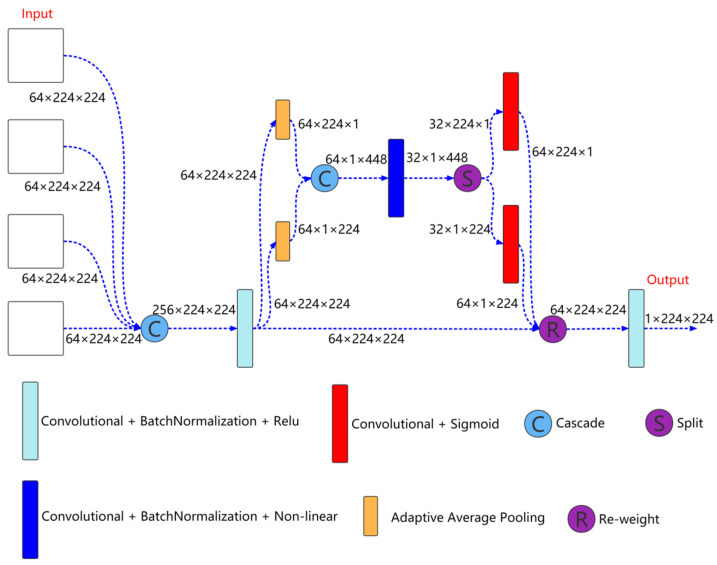
Output feature selection module structure.

Output feature selection module is shown in Figure 4, which is mainly used to fuse the output information of different scales to better extract coarse-grained information and fine-grained information. As shown in Figure 4, the inputs to the module are the features come from the four decoder layers. First, the features come from the four decoder layers are cascaded, and then the features are extracted through a convolution unit. The expression is as follows:

(4) $$x_{c}=g\left(Cascade\left(D_{0},\;D_{1},\;D_{2},\;D_{3}\right)\right)$$

Here, $x_{c}$ is the feature after convolution. $D_{0},\;D_{1},\;D_{2}$, and $D_{3}$ represents the features of the decoder layer 0, 1, 2, and 3, respectively. g() is the convolution block.

Then, the cascaded feature $x_{c}$ is input to the coordinate attention for further feature extraction,

(5) $$x^{CA}=CA\left(x_{c}\right)$$

Here, $x^{CA}$ is the feature further extracted by coordinate attention.

Finally, $x^{CA}$ is input to a convolution unit for feature extraction to obtain the feature finally used for segmentation prediction,

(6) $$x^{DO}=Convolution\left(x^{CA}\right)$$

Here, $x^{DO}$ is the feature finally used for segmentation prediction.

## 3. Experiments

### 3.1. Datasets, Implementation Details, and Evaluation Metrics

#### 3.1.1. Datasets

In our experiments, we use the ACDC [36] and the 2018 ASC [37]. The ACDC includes 100 3D cardiac MRI with physician annotated ground truth (right ventricle (RV), myocardium (Myo), and left ventricle (LV)). Same as TransUNet [30], we also divide these 100 3D cardiac MRI into training set, validation set, and test set according to the ratio of 7:1:2. The 2018 ASC includes 154 3D cardiac MRI with physician annotated ground truth (left atrium (LA)). We divide these 154 3D cardiac MRI into training set, validation set, and test set according to the ratio of 7:1:2.

Before using these datasets for model training, we normalize (0–1) each slice.

(7) $$y=\frac{x-Min}{Max-Min}$$

Here, *x* represents the original value before normalization, and *y* represent the normalized value. *Min* and *Max* represent the maximum and minimum values of the slice, respectively.

#### 3.1.2. Implementation Details

Our approach is implemented in Python with PyTorch and run on four RTX 3090 card. Our convolution block adopts VGG convolution block. It consists of two convolutional layers in series. Each convolutional layer consists of a 3 × 3 convolution, a normalization and a Relu activation function. The size of the input image with the largest resolution is 224 × 224. The input images of other resolutions are obtained by down sampling the input image with the largest resolution. We train our network in a deep supervision way, that is, predict and supervise the results at each decoder layer, and we take the output of the output feature selection module as our final prediction result. All models are trained with Adam optimizer with batch size 24, learning rate 5 × 10^4^, momentum 0.9, weight decay 1 × 10^4^ and max-epoch 1000. For ACDC, early stopping is set to 20. For 2018 ASC, early stopping is set to 10.

The loss function used in each method is the combination of binary cross entropy and dice loss.

#### 3.1.3. Evaluation Metrics

We measure the accuracy of segmentation by dice score (Dice), precision (Precision), and recall (Recall),

(8) $$\mathrm{Dice}=\frac{2\left(A\cap B\right)}{A\cup B}$$

(9) $$\mathrm{Precision}=\frac{\mathrm{TP}}{\mathrm{TP}+\mathrm{FP}}$$

(10) $$\mathrm{Recall}=\frac{\mathrm{TP}}{\mathrm{TP}+\mathrm{FN}}$$

Here, A is the segmentation result of the method, and B is the ground truth. The TP, FP, and FN represents the case numbers of true positives, false positives, and false negatives, respectively.

### 3.2. Ablation Experiments and Analyses

We analyze the influence of different components in the network on the average segmentation accuracy of the ACDC. The compared architectures include

(a) UNet + ViT as encoder (TransUNet),
(b) UNet + CvT as encoder (U + CvT),
(c) UNet + CvT as encoder + multiresolution aggregation module (U + CvT + MRAM),
(d) UNet + CvT as encoder + multiresolution aggregation module + output feature selection module (U + CvT + MRAM + OFSM).

In order to exclude the interference of random factors, we run each method 10 times to obtain the average value. The results are shown in Table 1. As shown in Table 1, compared with ViT, CvT is more conducive to the improvement of medical image segmentation performance. Our proposed MRAM and OFSM are effective in improving the performance of medical image segmentation.

### 3.3. Comparison with State-Of-The-Art Works and Discussion

#### 3.3.1. Comparison with State-Of-The-Art Works

**Figure 5:**
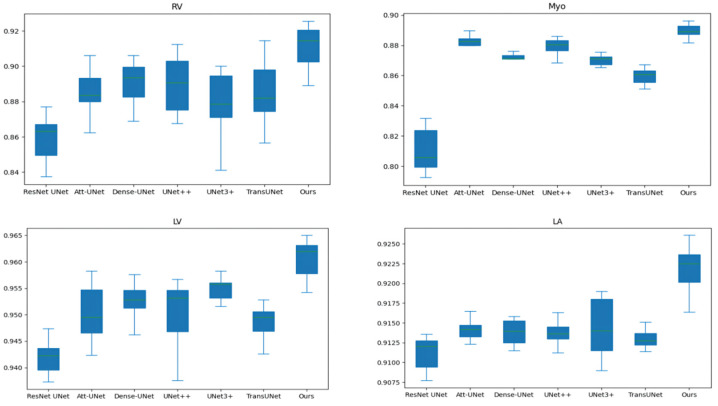
The box and whisker plot on the right ventricle (RV), myocardium (Myo), left ventricle (LV) and left atrium (LA).

Table 2 and Table 3 compares our results to state-of-the-art (SOTA) methods: ResNet UNet [17], Att-UNet [18], Dense-UNet [19], UNet++ [21], UNet3+ [23], TransUNet [30], Swin-UNet [31], and nnFormer [34]. In order to exclude the interference of random factors, we run each method 10 times to obtain the average value. Figure 5 shows the box and whisker plot on the right ventricle (RV), myocardium (Myo), left ventricle (LV), and left atrium (LA). As shown in Table 2 and Table 3 and Figure 5, our method outperforms TransUNet on all performance metrics, further demonstrating the effectiveness of our proposed method. In addition, our method achieves the best performance on most performance metrics.

**Figure 6:**
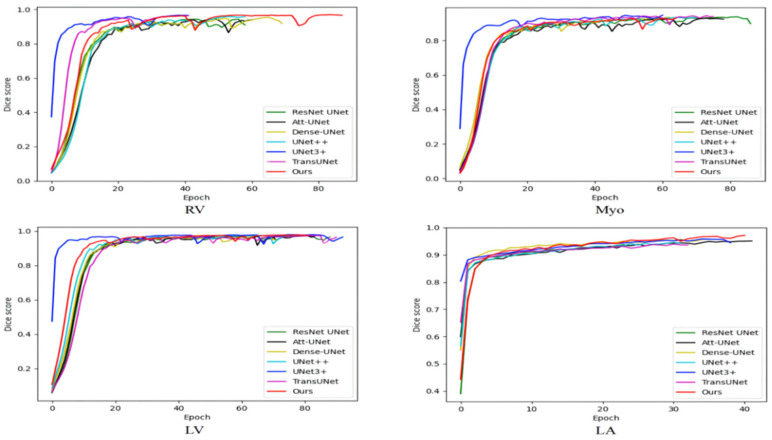
The variation of the training set dice score with iterations.

Table 4 compares the average training time of various methods on the ACDC and the 2018 ASC. As shown in Table 4, the number of parameters of our method is not particularly large, but the training time is longer than other methods because our method has more skip connections and is more difficult to train. Medical image segmentation does not require high real-time performance, and our method has a certain improvement in segmentation performance compared with TransUNet. Therefore, our method has certain practicability. Figure 6 shows the variation of the training set dice score with iterations. The ACDC is small and the model is prone to overfitting. Therefore, the training set dice score is not as large as possible, but some fluctuations are better, which can jump out of the local optimum. The 2018 ASC is large and the model is not prone to overfitting. Therefore, the larger the training set dice score, the stronger the model fitting ability and the better the performance. The training set dice score of our model on the ACDC has large fluctuations, indicating that our model has a good ability to jump out of the local optimum. The training set dice score is large on the 2018 ASC, indicating that our model has good fitting performance. On the whole, our model can balance the fitting performance and generalization performance and achieve relatively good comprehensive performance.

**Figure 7:**
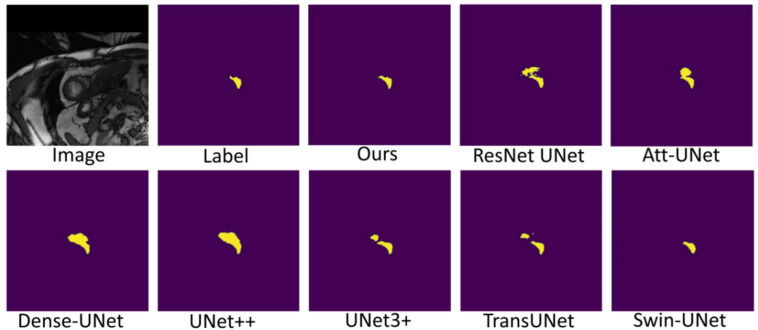
Comparison of right ventricle (RV) segmentation results.

**Figure 8:**
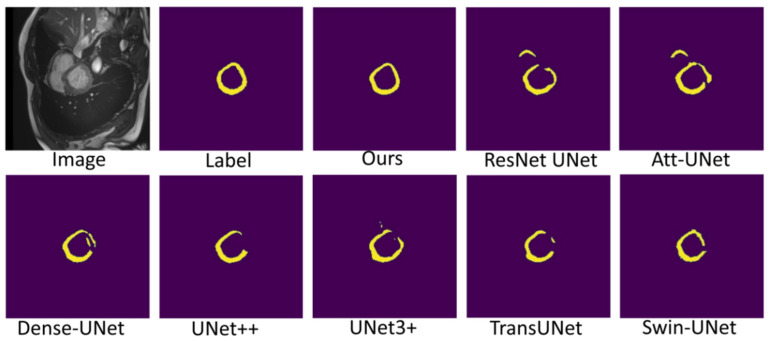
Comparison of myocardium (Myo) segmentation results.

**Figure 9:**
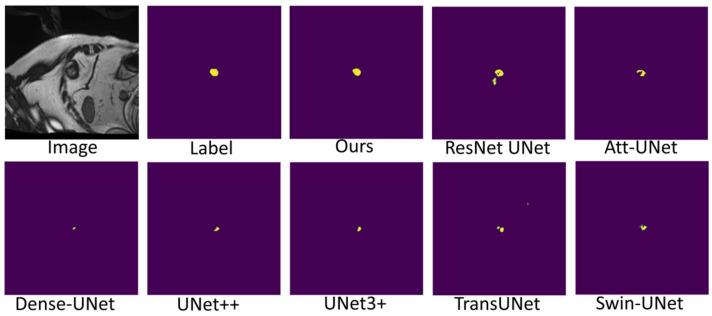
Comparison of left ventricle (LV) segmentation results.

Figure 7, Figure 8 and Figure 9 shows the visualizations on the right ventricle (RV), myocardium (Myo) and left ventricle (LV), respectively. As shown in Figure 7, our proposed method correctly segmented the clearly visible right ventricle and significantly reduced right ventricle mispredictions. Myocardium is a difficult tissue to segment; it is a circle on most slices. As shown in Figure 8, the segmentation results of other methods do not form a complete circle; only our method accurately predicts the result and forms a complete circle. The left ventricle is the tissue that is easier to segment. As shown in Figure 9, the segmentation results of other methods still have some mispredictions for left ventricle segmentation, and our method segmented the left ventricle perfectly.

#### 3.3.2. Discussion

Our method differs from current state-of-the-art methods mainly in that we leverage multiresolution image inputs to improve the encoder’s extraction of global and local features. High-resolution images are mainly used to extract local features, and low-resolution images are mainly used to extract global features. Then, we use a multiresolution aggregation module to fuse global and local features. As shown in Figure 7, Figure 8 and Figure 9, our method can locate the tissue accurately, but the segmentation accuracy of the edges is not high. This is probably because our low-resolution image is obtained by downsampling, and a lot of information may be lost during downsampling.

Regarding future improvements, there are mainly the following points:

(1) The multiresolution input image of our method shares the encoder, and the encoder may be difficult to balance the extraction of global and local features. Whether the multibranch encoding network is beneficial to improve feature extraction remains to be seen.
(2) Our method only fuses the features extracted from input images of different resolutions at the encoder side without considering the fusion at the decoder side.

## 4. Conclusions

In this paper, a multiresolution aggregation transformer UNet (MRA-TUNet) for medical image segmentation is proposed. The input features of the network are enhanced by fusing the input image information of different resolutions through a multiresolution aggregation module. The output feature selection module is used to fuse the output information of different scales to better extract coarse-grained information and fine-grained information. In addition, we try to introduce a coordinate attention structure for the first time to further improve the segmentation performance. We compare with state-of-the-art medical image segmentation methods on the automated cardiac diagnosis challenge and the 2018 atrial segmentation challenge. The experimental results on two datasets show that our method outperforms eight state-of-the-art medical image segmentation methods in dice score, precision, and recall.

## Figures and Tables

**Table 1:** Ablation analysis on ACDC for different components in the network. All methods were run 10 times to take the average. The best performance is shown in red.

	Dice Average	Precision Average	Recall Average
TransUNet	0.898	0.885	0.923
U + CvT	0.909	0.901	0.926
U + CvT + MRAM	0.915	0.910	0.926
U + CvT + MRAM + OFSM	0.921	0.910	0.933

**Table 2:** Comparison with state-of-the-art methods on the ACDC. All methods were run 10 times to take the average and standard deviation (average ± standard deviation). The best performance is shown in red (the data of Swin-UNet and nnFormer are from the corresponding original literature, and the data of other methods are that we train under the same conditions).

Methods	Dice			Precision			Recall		
	RV	Myo	LV	RV	Myo	LV	RV	Myo	LV
ResNet UNet [17]	0.859 ± 0.012	0.810 ± 0.013	0.942 ± 0.003	0.843 ± 0.033	0.848 ± 0.016	0.940 ± 0.009	0.913 ± 0.019	0.812 ± 0.037	0.957 ± 0.009
Att-UNet [18]	0.885 ± 0.012	0.881 ± 0.007	0.949 ± 0.008	0.861 ± 0.019	0.876 ± 0.012	0.950 ± 0.012	0.929 ± 0.014	0.895 ± 0.010	0.957 ± 0.017
Dense-UNet [19]	0.891 ± 0.012	0.869 ± 0.007	0.953 ± 0.003	0.858 ± 0.014	0.869 ± 0.016	0.943 ± 0.009	0.939 ± 0.009	0.879 ± 0.015	0.969 ± 0.004
UNet++ [21]	0.885 ± 0.022	0.880 ± 0.005	0.951 ± 0.006	0.873 ± 0.038	0.870 ± 0.010	0.949 ± 0.011	0.914 ± 0.026	0.898 ± 0.013	0.964 ± 0.005
UNet3+ [23]	0.878 ± 0.019	0.870 ± 0.003	0.955 ± 0.003	0.847 ± 0.021	0.881 ± 0.008	0.951 ± 0.009	0.920 ± 0.024	0.867 ± 0.011	0.962 ± 0.008
TransUNet [30]	0.885 ± 0.016	0.860 ± 0.005	0.949 ± 0.003	0.849 ± 0.031	0.861 ± 0.016	0.946 ± 0.007	0.939 ± 0.008	0.870 ± 0.017	0.958 ± 0.006
Swin-UNet [31]	0.886	0.857	0.958	-	-	-	-	-	-
nnFormer [34]	0.902	0.895	0.956	-	-	-	-	-	-
Ours	0.911 ± 0.012	0.890 ± 0.004	0.961 ± 0.004	0.882 ± 0.026	0.889 ± 0.016	0.959 ± 0.008	0.944 ± 0.019	0.890 ± 0.019	0.964 ± 0.014

**Table 3:** Comparison with state-of-the-art methods on the 2018 ASC. All methods were run 10 times to take the average and standard deviation (average ± standard deviation). The best performance is shown in red. (the data of all methods are that we train under the same conditions, and the standard deviation of Swin-UNet is not available).

Methods	Dice	Precision	Recall
	LA	LA	LA
ResNet UNet [17]	0.911 ± 0.002	0.910 ± 0.010	0.921 ± 0.009
Att-UNet [18]	0.914 ± 0.002	0.911 ± 0.008	0.924 ± 0.008
Dense-UNet [19]	0.914 ± 0.002	0.909 ± 0.004	0.925 ± 0.004
UNet++ [21]	0.914 ± 0.002	0.914 ± 0.007	0.921 ± 0.007
UNet3+ [23]	0.915 ± 0.004	0.921 ± 0.007	0.916 ± 0.009
TransUNet [30]	0.913 ± 0.002	0.904 ± 0.008	0.928 ± 0.006
Swin-UNet [31]	0.909	0.901	0.924
Ours	0.923 ± 0.003	0.919 ± 0.007	0.927 ± 0.008

**Table 4:** Compares the average training time of various methods on the ACDC and 2018 ASC. The best performance is shown in red.

Methods	Average Training Time (s)		Parameters (Million)
	ACDC	2018 ASC	
ResNet UNet [17]	554	5308	82
Att-UNet [18]	609	6268	35
Dense-UNet [19]	544	4359	2
UNet++ [21]	1225	13,562	36
UNet3+ [23]	977	10,857	26
TransUNet [30]	448	5762	105
Ours	1175	12,891	56

## Data Availability

The data used to support the findings of this study are available from the corresponding author upon request.
